# Sex differences in ischemic heart disease and heart failure biomarkers

**DOI:** 10.1186/s13293-018-0201-y

**Published:** 2018-09-17

**Authors:** Kimia Sobhani, Diana K. Nieves Castro, Qin Fu, Roberta A. Gottlieb, Jennifer E. Van Eyk, C. Noel Bairey Merz

**Affiliations:** 1 Pathology and Laboratory Medicine, Cedars-Sinai Medical Center, Los Angeles, CA USA; 2 Barbra Streisand Women’s Heart Center, Cedars-Sinai Smidt Heart Institute, 127 S. San Vicente Blvd, Suite A3206, Los Angeles, CA 90048 USA; 3 Advanced Clinical Biosystems Research Institute, Cedars-Sinai Medical Center, Los Angeles, CA USA

**Keywords:** Sex, Cardiovascular, Biomarker

## Abstract

Since 1984, each year, more women than men die of ischemic heart disease (IHD) and heart failure (HF), yet more men are diagnosed. Because biomarker assessment is often the first diagnostic employed in such patients, understanding biomarker differences in men vs. women may improve female morbidity and mortality rates.

Some key examples of cardiac biomarker utility based on sex include contemporary use of “unisex” troponin reference intervals under-diagnosing myocardial necrosis in women; greater use of hsCRP in the setting of acute coronary syndrome (ACS) could lead to better stratification in women; and greater use of BNP with sex-specific thresholds in ACS could also lead to more timely risk stratification in women.

Accurate diagnosis, appropriate risk management, and monitoring are key in the prevention and treatment of cardiovascular diseases; however, the assessment tools used must also be useful or at least assessed for utility in both sexes. In other words, going forward, we need to evaluate sex-specific reference intervals or cutoffs for laboratory tests used to assess cardiovascular disease to help close the diagnostic gap between men and women.

## Background

Since 1984, each year, more women than men die of cardiovascular disease (CVD) [1], yet more men than women are diagnosed [2]. While overall female CVD death rates began to decline in the 2000s [3], there continues to be both an excess of female deaths and many remaining unknowns as to the etiology of sex-based differences. Ischemic heart disease (IHD) is the leading cause of death for women in the USA, killing 289,758 women in 2013—which equates to approximately *1 in every 4* female deaths [4]. Heart failure with preserved ejection fraction (HFpEF) is a condition which predominantly impacts women, is currently poorly understood, and lacks evidence-based therapy [5]. The aging of the baby boomer population combined with female longevity is a convenient but inaccurate explanation for these sex differences, because CVD death rates have actually declined in older women concomitant with the declines for both older and younger men, compared to an *increase* in younger women [6]. Recent work suggests that there may be a diagnosis gap, whereby relatively more men are diagnosed and treated for IHD and HF compared to women, potentially accounting in part for increased female mortality [7].

Prior work suggests that under-diagnosis of IHD in women could be due to sex differences in phenotypic presentation, i.e., the signs and symptoms of IHD in men vs. women [8]. IHD and HF are diagnosed by a combination of (1) clinical presentation, (2) circulating biomarker levels, and (3) imaging. IHD symptoms appear to differ between women and men and also by ethnicity, which can contribute to a failure to proceed with appropriate diagnostic tests and procedures. Specifically, women with acute coronary syndrome (ACS) more often present with atypical symptoms such as jaw pain and nausea compared with men [9], and non-Caucasian ethnicities are associated with higher rates of atypical angina symptoms more often ascribed to digestive rather than cardiac etiology [10]. Because biomarker assessment is a more specific and often the first diagnostic measure employed in patients with suspected CVD, understanding cardiac biomarker differences in men vs. women may improve female CVD morbidity and mortality rates.

In 2001, a National Institutes of Health (NIH) working group standardized the definition of a biomarker as “a characteristic that is objectively measured and evaluated as an indicator of normal biological processes, pathogenic processes, or pharmacologic responses to a therapeutic intervention” and also defined types of biomarkers based on the information they provide [11]. Table 1 provides definitions adapted from the NIH working group document.

**Table 1:** Biomarkers: a basic glossary (derived from NIH working group) [11]

Biological marker (biomarker):	A characteristic that is objectively measured and evaluated as an indicator of normal biological processes, pathogenic processes, or pharmacological responses to a therapeutic intervention.
Type 0 biomarker:	A marker of the natural history of a disease and correlates longitudinally with known clinical indices.
Type I biomarker:	A marker that captures the effects of a therapeutic intervention in accordance with its mechanism of action.
Surrogate endpoint (type 2 biomarker):	A marker that is intended to substitute for a clinical endpoint; a surrogate endpoint is expected to predict clinical benefit (or harm or lack of benefit or harm) on the basis of epidemiological, therapeutic, pathophysiological, or other scientific evidence.
Risk factor:	A risk factor is associated with a disease because it is in the causal pathway leading to the disease.
Risk marker:	A risk marker is associated with the disease (statistically) but need not be causally linked; it may be a measure of the disease process itself.
Clinical endpoint:	A characteristic or variable that reflects how a patient feels, functions, or survives.
Intermediate (non-ultimate) endpoint:	A true clinical endpoint (a symptom or measure of function, such as symptoms of angina frequency or exercise tolerance) but not the ultimate endpoint of the disease, such as survival or the rate of other serious and irreversible morbid events.
Validation of a biomarker (assay or method validation):	A process for assessing performance characteristics (i.e., sensitivity, specificity, and reproducibility) of a biomarker measurement or an assay technique.
Qualification of a biomarker (clinical validation):	The evidentiary process linking a biomarker to disease biology or clinical outcome.
Evaluation of a biomarker:	A process of linking biomarkers to outcomes, often with a view to establish surrogate status.

Several clinically established cardiovascular circulating biomarkers are measured in order to help diagnose, stratify risk, and monitor people with suspected ACS, acute myocardial infarction (MI), and HF [12]. Use of one or more of these biomarkers can help physicians identify an IHD and/or HF condition and initiate appropriate therapy, as well as follow the course of disease.

In 2007, the National Academy of Clinical Biochemistry (NACB) and International Federation of Clinical Chemistry (IFCC) committee recommended that sex-specific reference ranges should be developed and utilized in clinical practice for some cardiac biomarkers [13], yet common laboratory practice for most of these biomarkers still revolves around the use of universal ranges or cutoffs for both men and women. As mentioned earlier, lack of sex-specific cardiac biomarker thresholds in men and women may contribute to IHD and HF under-diagnosis in women and potentially increased morbidity and mortality as a result, or conversely an over-diagnosis in men. Additionally, coronary microvascular dysfunction appears to be more prevalent in women than in men and may result in part from hormonal and immunomodulatory differences in men and women, which further supports a need to define sex-specific measures and thresholds when studying cardiovascular disease [14–16].

We review here literature that highlights sex differences in IHD and HF biomarkers and also discuss instances where knowledge of sex differences is lacking and likely warranted.

## Methods

**Fig. 1:**
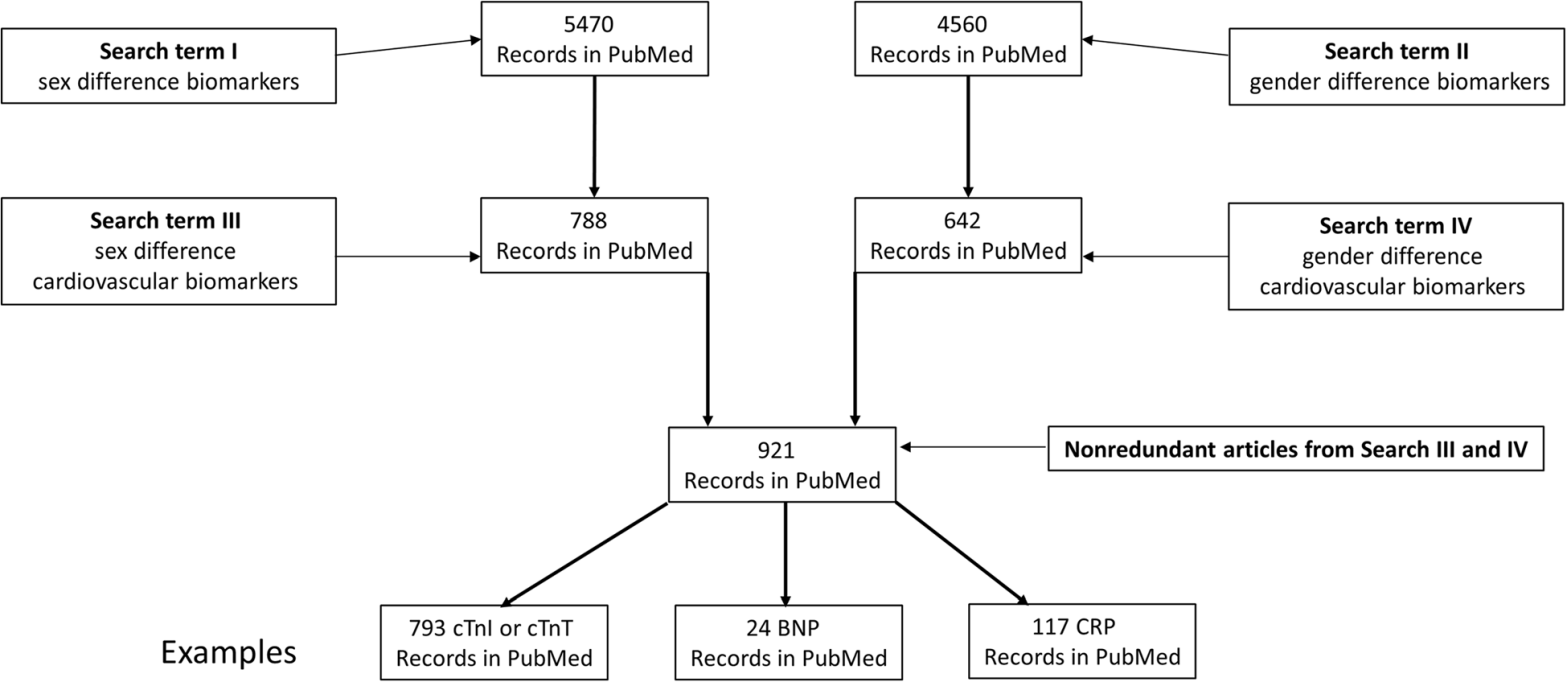
Schema of database searches on gender and sex-related biomarkers with search terms specified. Results for PubMed are shown as an example. This search was conducted in 2016

Table 2 lists cardiac circulating biomarkers, including markers of inflammation and atherosclerosis, endothelial function, thrombosis, oxidative stress, ischemia/necrosis, hemodynamic stress, HF mortality, renal dysfunction, metabolic dysfunction/lipid dysregulation, and brain injury, used in our literature search spanning from the year 2000 to approximately through 2016. We searched the electronic databases of PubMed and MEDLINE via OVID using the aforementioned biomarker categories as keywords with sex or gender differences included. We did not impose any restrictions regarding language, publication date, or study setting. Data and evidence-based statements were extracted from each paper, and relevant references of retrieved articles were used as additional references. The process for these searches is shown in Fig. 1.

**Table 2:** Cardiac biomarkers (modified with permission) [67]

Biomarkers	Biology	Conditions associated with elevations
Biomarkers of inflammation and atherogenesis		
hs-CRP	hs-CRP is an acute phase protein produced predominantly by hepatocytes. Elevations are associated with an increased risk for coronary artery disease and ACS, but it is unclear if it is causally related.	ACS
Metalloproteinases (MMP-9, MMP-11)	Metalloproteinases (MMP-9, MMP-11) are proteases produced by fibroblasts, osteoblasts, and vascular smooth muscle cells. They are highly expressed in unstable plaques.	ACS
PAPP-A	PAPP-A (pregnancy-associated plasma protein A) is a high molecular weight, zinc-binding metalloproteinase that is associated with vulnerable plaque and may predict cardiovascular disease and mortality.	ACS
Cathepsin S	Cathepsin-S promotes intra- and extra-cellular proteolysis and is associated with the development of cardiovascular disease and cancer.	CAD
IL-1β	IL-1β is an inflammatory cytokine involved in myocardial remodeling post-MI and in CHF.	ACS, CHF
IL-1Ra	IL-1Ra is inactive protein that binds to the IL-1 receptor, functioning as antagonist to IL-1 (functionally as an anti-inflammatory protein).	ACS
IL-6	IL-6 is an interleukin that acts as both an inflammatory and anti-inflammatory cytokine. IL-6 is also considered a myokine, a cytokine produced by muscle, and is secreted in response to muscle contraction. Increased IL-6 has been associated with decreased cardiac functional status in CHF.	CHF, AFIB, ischemia, CV risk
Chemotactic molecules (MCP-1, CCR1, CCR2)	CCR2 and CCR5 are two CC chemokine receptors. Chemokines are the main modulators of inflammatory and repair processes. CCR1, in particular, may have a key role in heart repair after MI. MCP-1 (monocyte chemotactic protein-1) plays a critical role in the development of cardiovascular diseases.	CAD, ACS
Myeloperoxidase	Myeloperoxidase is a leukocyte-derived enzyme that catalyzes the formation of reactive oxidants and may promote plaque formation and rupture.	ACS, CHF
Neopterin	Neopterin is a marker of macrophage/monocyte activation. It is associated with atherosclerosis, plaque instability, and CHF. Increased levels may serve as predictors of future cardiovascular events.	CAD, ACS, CHF
Growth differentiation factor-15	Growth differentiation factor-15 is a member of the transforming growth factor beta (TGFβ) superfamily. Produced by cardiomyocytes, activated macrophages, endothelial cells, vascular smooth muscle cells, and adipocytes, it regulates inflammatory and apoptotic pathways needed for development, differentiation, and tissue repair. It is upregulated in many tissues following injury, ischemia, and other forms of stress.	CAD, ACS
Placental growth factor vascular endothelial growth factor	Placental growth factor is a member of the vascular endothelial growth factor family (VEGF). Originally described in the placenta, it recruits macrophages into atherosclerotic lesions, stimulates pathological angiogenesis, and is associated with worse prognosis in ACS.	CAD, ACS
Markers of fibrosis (galectin-3)	Galectin 3 is a β-galactoside-binding protein expressed by a number of cell types, including neutrophils and macrophages. In the heart, levels are almost undetectable in cardiomyocytes, whereas cardiac fibroblasts express higher levels. It is thought to represent a link between inflammation and fibrosis. Elevated Gal-3 is associated with increased CHF and mortality risk.	CHF
Fetuin-A	Fetuin-A is a hepatic secretory protein that inhibits arterial calcification.	CAD
Vascular calcification (osteoprogeterin) osteoprotegerin	Osteoprotegerin (OPG) is a glycoprotein member of the tumor necrosis factor receptor superfamily that acts as a decoy receptor for receptor activator of nuclear factor κB ligand (RANKL) and tumor necrosis factor-related apoptosis-inducing ligand (TRAIL). Elevated OPG is associated with coronary and peripheral artery disease and subclinical atherosclerosis, and coronary artery calcium, although its role in atherosclerotic calcification remains speculative. It is associated with vascular mortality and has been reported to be higher in patients with acute MI.	CAD ACS, CAD
Myeloid-related proteins 8/14 (MRP8/14)	Myeloid-related proteins − 8 and − 14 are calcium-binding proteins expressed and secreted by neutrophils, monocytes, and subsets of macrophages. They are a useful biomarker of disease activity in inflammatory disorders. Increased levels are associated with increased cardiovascular events.	ACS
Biomarkers of endothelial function		
E-selectin	E-selectin is an endothelial cell adhesion molecule expressed only on endothelial cells activated by cytokines. Like other selectins, it plays an important part in inflammation, recruiting leukocytes to the site of injury. E-selectin and PECAM-1 may play an important role in inflammatory reaction and development of vulnerable plaque.	ACS, CAD
Pentraxin 3 (PTX3)	PTX3 is released as a response to vascular damage and therefore may provide more information on development and progression of atherosclerosis than other less specific markers such as CRP.	CAD
VCAM-1	VCAM-1 (vascular cell adhesion molecule-1) binds monocytes and T lymphocytes, the types of leukocytes found in early atherosclerotic plaques. It also functions in leukocyte-endothelial cell signal transduction, and it may play a role in the development of atherosclerosis.	CAD
ICAM-1	ICAM-1 (intercellular adhesion molecule-1) may play a role in signal transduction associated primarily with proinflammatory pathways. In particular, ICAM-1 signaling seems to produce a recruitment of inflammatory immune cells such as macrophages and granulocytes.	CAD
Biomarkers of thrombosis		
vWF	vWF (von Willebrand factor) is a large multimeric glycoprotein present in blood plasma and produced by Weibel-Palade bodies in endothelium, megakaryocytes, platelet α-granules, and subendothelial connective tissue. VWF plays a pivotal role in platelet adhesion and aggregation at sites of high shear rates (e.g., in coronary arteries that have stenotic or ruptured atherosclerotic plaque lesions) in patients with pre-existing vascular disease.	CAD, ACS
Tissue factor	Tissue factor (TF) TF is the cell surface receptor for the serine protease factor VIIa. The best known function of TF is its role in blood coagulation. The complex of TF with factor VIIa catalyzes the conversion of the inactive protease factor X into the active protease factor Xa. TF is expressed by cells which are normally not exposed to flowing blood such as smooth muscle cells and cells surrounding blood vessels (e.g., fibroblasts). This can change when the blood vessel is damaged by physical injury or rupture of atherosclerotic plaques.	ACS, invasive cardiology
sCD40L	Soluble CD40 ligand (sCD40L) is contained in platelet granules, and thus, its presence in the blood is a marker of platelet activation. By interacting with CD40, which is found on endothelial and smooth muscle cells, sCD40L may trigger the release of inflammatory mediators, lead to increased activity of matrix metalloproteinases, and activate the coagulation cascade. sCD40L is an independent predictor of ACS outcomes.	ACS
Prothrombin fragment 1.2	Prothrombin fragment 1.2 (F1.2) is an activation peptide generated during the conversion of prothrombin to thrombin. As a marker of thrombin generation, F1.2 has clinical potential in assessing thrombotic risk.	ACS, CAD
Thrombin precursor protein	Thrombus precursor protein (TpP) is a biomarker that is used to measure soluble fibrin polymers, which are the penultimate products in fibrin formation. In patients with ACS, increased levels of TpP are associated with an increased risk of death or ischemic complications.	ACS
D-dimer	D-dimer is a fibrin degradation product (FDP), a small protein fragment present in the blood after a blood clot is degraded by fibrinolysis. It is an excellent marker of fibrinolytic activity.	ACS
Biomarkers of oxidative stress		
Lp-PLA2	Lp-PLA2 is a protein produced by inflammatory cells. It circulates mainly with LDL (< 20% is associated with HDL or remnant lipoproteins), and it is responsible for hydrolyzing oxidized phospholipids in LDL. It is highly upregulated in atherosclerotic plaques and may be directly involved in development of atherosclerosis and plaque rupture.	ACS, CAD
Oxidized amino acids	Amino acids are the building blocks of protein, and oxidative stress plays a central role in the pathogenesis of diverse chronic inflammatory disorders including cardiovascular disease. For example, elevated levels of homocysteine, an amino acid in the blood, increase the oxidation of LDL cholesterol which in turn contributes to the formation of fatty deposits along the arterial walls leading to atherosclerosis.	CAD
Oxidized apoA1	Apolipoprotein A1 (apoA1) is a major protein component of high-density lipoprotein, or HDL cholesterol. Oxidized HDL impairs cholesterol efflux from monocytes and macrophages and may result in a loss of anti-inflammatory effects and a resulting change to a proinflammatory state.	CAD
ADMA and other arginine metabolism products	Asymmetric dimethylarginine (ADMA), a naturally occurring chemical found in blood plasma, is an endogenous nitric oxide synthase inhibitor that has been linked to cardiovascular risk. It is closely related to L-arginine, a conditionally essential amino acid. ADMA interferes with L-arginine in the production of nitric oxide, a key chemical involved in normal endothelial function.	CAD
Secretory phospholipase A2	SPLA2 is a member of a family of enzymes that catalyze the breakdown of phospholipids into fatty acids and lysophospholipids, thereby contributing to cholesterol loading of macrophages, and activation of inflammatory and atherogenic pathways.	CAD
Biomarkers of ischemia/necrosis		
Creatine kinase-MB	The MB fraction (or isoenzyme) is the most clinically used assays for CK-MB and is increased with myocardial necrosis; however, it is not as specific for ACS or other myocardial damage as hstroponin.	MI, CHF, CAD
High-sensitivity cardiac troponin	High-sensitivity cardiac troponin is increased with myocardial necrosis; however, it is not specific to ACS as it can be increased in any condition that results in myocardial damage, including CHF, pulmonary embolism, or severe HTN.	MI, CHF, CAD
Malondialdehyde-modified low-density lipoprotein	MDA-modified low density lipoprotein plays an important role in the development of atherosclerosis as its uptake by macrophages and smooth muscle cells leads to formation of foam cells. MDA-modified LDL has a diagnostic accuracy as an independent biochemical marker for atherosclerosis.	CAD
Fatty acid binding protein	Heart-type fatty acid-binding protein (H-FABP) is a small cytoplasmic protein (15 kDa) released from cardiac myocytes following an ischemic episode. It is an early biomarker for myocardial infarction, detected in the blood within 1 to 3 h of ACS.	ACS
Biomarkers of hemodynamic stress		
BNP and/or NT-proBNP	The concentration of B-type natriuretic peptide (BNP) or N-terminal pro-B-type natriuretic peptide (NT-proBNP) are increased with myocardial (ventricular, and to a lesser extent, atrial) stretch. Both peptides are accepted markers for cardiac dysfunction. Elevated levels of either of these peptides are associated with and are equally useful as an aid in the diagnosis of CHF.	CHF
Copeptin	Copeptin is the stable C-terminal part of pro-arginine-vasopressin (AVP). It is released with AVP after hemodynamic or osmotic stimuli and is also an endocrine stress hormone. It is released into the circulation early after MI onset and may aid in rapid diagnosis.	ACS, CHF
Mid-region pro-adrenomedullin	Mid-region pro-adrenomedullin causes vasodilation via stimulation of nitric oxide production and is upregulated in CHF as a compensatory mechanism for the associated hemodynamic abnormalities.	CHF
Urocortin-1	Urocortin-1 is a vasoactive member of the corticotropin-releasing factor family. Lower levels provoke increases in heart rate, cardiac output, and coronary blood flow; at very high concentrations, it causes vasodilation and a decline in total peripheral resistance.	CHF
Arginine vasopressin	Arginine vasopressin (AVP) is an antidiuretic and vasoconstrictive hormone that is released from the hypothalamus in response to changes in plasma osmolality and hypovolemia and has two principal sites of action: kidneys and blood vessels. AVP is upregulated in CHF.	CHF
Endothelin-1	Endothelin-1 is produced by the endothelium in response to angiotensin II, inflammatory mediators, and vascular shear stress. It is responsible for vasoconstriction, activation of reactive oxygen species, and ventricular remodeling.	CHF, HTN
Biomarkers of heart failure mortality		
ST-2	ST-2 is an interleukin-1 receptor family member expressed as both transmembrane (ST2L) and soluble (sST2) isoforms. Plasma levels of sST2 are elevated in inflammatory diseases such MI and heart failure, as well as COPD, pneumonia, and sepsis.	MI, CHF
Biomarkers of renal dysfunction		
Cystatin-C	Cystatin-C is a cysteine protease inhibitor synthesized by all nucleated cells in the body. It is freely filtered by the glomerulus, reabsorbed completely, and is not secreted. A rise in serum cystatin C is a marker of renal dysfunction and may have a role as a prognostic marker in patients with coronary artery disease and heart failure as it appears to have additive prognostic value to creatinine.	Acute kidney injury, CAD, CHF
Neutrophil gelatinase-associated lipocalin (NGAL)	Neutrophil gelatinase-associated lipocalin (NGAL) is highly upregulated at an early stage of renal injury and can be rapidly detected in the urine.	Acute kidney injury, CHF
KIM-1	KIM is a type 1 transmembrane glycoprotein not detectable in normal kidney tissue or urine, but it is expressed at very high levels in dedifferentiated proximal tubule epithelial cells in kidneys and appears in urine after ischemic or toxic injury.	Acute kidney injury
Biomarkers of metabolic/lipid dysregulation		
Adiponectin	Adiponectin is an adipokine protein hormone that modulates a number of metabolic processes, including glucose regulation and fatty acid oxidation. A low level of adiponectin is an independent risk factor for developing metabolic syndrome or diabetes mellitus. Low levels of adiponectin are associated with obesity-linked cardiovascular diseases, including ischemic heart disease and peripheral artery disease.	CAD, MetS
Leptin	Leptin is a 16-kDa protein hormone that plays a key role in regulating energy intake and energy expenditure, including appetite/hunger and metabolism. In the absence of established CHD, the association between obesity and CHF may be mediated by plasma leptin. In those with CHD, obesity appears to increase the risk of CHF independent of leptin.	CHF
Resistin	Resistin (an adipokine) also known as adipose tissue-specific secretory factor (ADSF) or C/EBP-epsilon-regulated myeloid-specific secreted cysteine-rich protein (XCP1) is a cysteine-rich protein encoded by the RETN gene. Resistin increases the production of LDL in human liver cells and also stimulates degradation of LDL receptors in the liver.	CHF, CAD
C-peptide	C-peptide is a cleavage product of proinsulin and is a marker of insulin production. C-peptide levels help to distinguish between type 1 and type 2 diabetes and to evaluate insulin resistance and hypoglycemia.	CHF
Phospholipid fatty acids (EPA and DHA)	EPA+DHA are the major fatty acids contributing to the total omega-3 fatty acids in human serum phospholipid. EPA and DHA omega-3 fatty acids are enriched in oily fish. In fatal ischemic heart disease, combined EPA+DHA levels comprising at least 4.6% of total fatty acids in serum were associated with 70% lower cardiovascular risk than those with lower levels of these fatty acids.	CAD
Apolipoprotein E	ApoE is synthesized by the liver as part of VLDL; it functions in the transport of triglycerides to the liver tissue. It is also incorporated into HDL (as HDL-E) and functions in cholesterol distribution among cells.	CAD
Cholesteryl ester transfer protein activity	Cholesteryl ester transfer protein (CETP), also called plasma lipid transfer protein, is a plasma protein that facilitates the transport of cholesteryl esters and triglycerides between the lipoproteins. Inhibiting CETP activity raises high-density lipoprotein cholesterol and may be cardioprotective; however, lower plasma CETP activity has also been associated with greater CVD risk.	CAD
Biomarkers of brain damage		
S100 beta	S100 calcium binding protein B (S100 Beta) is a protein of the S-100 protein family. S100 proteins are involved in the regulation of a number of cellular processes such as cell cycle progression and differentiation. In a recent study, in patients with cardiac arrests, patients with good outcomes had significantly lower S100B levels at all time points and lower neuron-specific enolase (NSE) levels on days 1 and 3 compared with those with poor outcomes.	ACS, CABG
Neuron-specific enolase	Neuron-specific enolase (NSE) is an enzyme used as a biomarker of hypoxic brain damage and may predict death or vegetative state in comatose cardiac-arrest survivors.	ACS

### Creatinine kinase MB

At one point in recent history, creatine kinase represented our most cardiac-specific biomarker, especially with development of creatine kinase isoenzyme assays in contrast to previously used (and extremely non-specific) analytes such as lactate dehydrogenase. Creatine kinase (CK) exists as isoenzymes with various combinations of muscle (CKM) and brain (CKB) subunits (i.e., MM, MB, BB). These isoenzymes facilitate the transfer of high-energy phosphates into and out of mitochondria and exist in many tissues including the heart, skeletal muscle, and brain. As a result of vigorous exercise, organ damage, and skeletal muscle disease, they can be nonspecifically elevated in plasma. The MB fraction (or isoenzyme) is most concentrated in the heart tissue, and most clinically applied assays for creatinine kinase MB (CK-MB) measure its mass, which is more sensitive than enzyme activity-based assays. CK-MB represented the “gold standard” for laboratory diagnosis of myocardial infarction (MI) through the late 80s and 90s. Additionally, mass assays for CK-MB mostly avoid picking up macrokinases (i.e., CK isoenzymes bound to IgG or aggregates of mitochondrial CK) that can prevent clear interpretability with activity-based assays. The presence of macrokinases should be considered as a possible cause of elevation when CK-MB is a very high percentage (e.g., > 20%) of total CK [17]. Furthermore, using criteria for CK-MB as fraction of total CK can lead to false elevations due to chronic skeletal muscle disease [18]. CK-MB can account for as much as 50% of total CK elevation with chronic skeletal muscle injury (i.e., dermatomyositis and polymyositis) due to increased production of the B chain of CK protein. An additional area of CK-MB utility is its correlation to infarct size through serial measurements; however, more recent comparisons with cardiac troponins suggest that troponins can still provide more accurate estimates [19, 20].

The criterion most commonly used for the diagnosis of acute MI was two serial elevations above the diagnostic cutoff level or a single result more than twice the upper limit of normal. Although CK-MB is approximately 3–4 times more concentrated in the myocardium, it also exists in the skeletal muscle and false-positive elevations occur in a number of clinical settings, including trauma, heavy exertion, and myopathy.

NACB and IFCC guidelines published in 2007 noted that CK-MB is the preferred *alternative* biomarker for MI, and many hospitals still use it in conjunction with troponin [13], although current American Heart Association/American College of Cardiology (AHA/ACC) guidelines for both non-ST segment elevation MI (NSTEMI) and ST segment elevation MI (STEMI) from 2014 and 2013, respectively, no longer support use of CK-MB for this application. Skeletal muscle is also composed of small amounts of CK-MB (1–3%) [21]. Since men on average have greater body muscle mass, they would be expected to have a higher concentration of this biomarker. Indeed, a sex difference was consistently demonstrated using seven different assays [Abbott AxSYM, Bayer Centaur, Beckman Access, Dade-Behring Dimension RxL, Vitros ECi, Roche, Tosoh AIA] where an over twofold higher 99th percentile for CK-MB was found for males vs. females [22]. Further investigation assessed two different analytical platforms [UniCel® DxI 800 and Access® 2] to establish sex-specific 99th percentiles for CK-MB mass and found values to be significantly higher in men than in women [23].

These data strongly suggest that much like troponins (as discussed in the next section), use of male CK-MB 99th percentile would be expected to under-diagnose myocardial necrosis in women. Indeed, Wiviott et al. suggest that women with high probability of ACS NSTEMI, but no biomarker elevation, could benefit from a multimarker approach [24].

### Troponins

Cardiac troponins (cTn) are muscle regulatory proteins that control the calcium-mediated interaction of actin and myosin (muscle contraction) and consist of cytosolic and structural pools, with most troponin present as structural proteins [25]. Cardiac troponin I (cTnI) and cardiac troponin T (cTnT) are the two protein subunits of the troponin tri protein-complex (troponin C being the third but is not cardiac specific), which are actually measured in plasma to assess cardiac damage. Both cTnI and TnT are independently measured using monoclonal antibodies in immunometric assay formats resulting in clinical assays specific (almost exclusively) to cardiac muscle damage. While they do not specifically identify the underlying cause of cell injury, they are considered to be the current gold-standard biomarker for myocardial injury and necrosis [13].

Cardiac troponin concentrations typically begin to rise 2–3 h after the onset of acute MI. At 2–3 h after initial presentation, up to 80% of patients with MI will have detectable troponin elevations. Markers that rise earlier than troponins, such as myoglobin and CK isoforms, actually have been shown to provide little additional diagnostic or clinical utility when a sufficiently analytically sensitive troponin assay (i.e., an assay with a coefficient of variation approaching 10% at the 99th percentile) is employed in the lab [26], although, in practice, desired precision at the 99th percentile is typically not achieved for existing (contemporary) US Food and Drug Administration (FDA) approved troponin assays that are in use. cTnI is considered specific to the heart muscle as no other isoform of this fraction has even been detected or discovered in other muscle tissue [27]; however, cTnT is believed to be potentially expressed to a very minor extent in the skeletal muscle, due to its previous measurement in patients with specific skeletal muscle myopathies [28]. TnI can also be mildly overexpressed in these rare patients, but the prevalence of elevated expression compared to TnT is much lower. Despite their current widespread use, contemporary cardiac troponin assays lack sex-specific reference value reporting, even for widely used commercial assays that indicate 99th percentile cutoffs or ranges 1.2–2.4-fold higher in males than females [29]. Healthy animal data demonstrates higher circulating cTnI concentrations in males compared to females of the same species [30].

Further investigation has evaluated sex differences in troponin and clinical manifestations of CVD. Säfström et al. evaluated exercise stress testing and cTnT in subjects with suspected myocardial ischemia [31]. The women studied were older and had higher incidence of CVD-related events and diagnoses; however, only 49% of the women compared to 69% of men had cTnT levels that met the threshold for MI (≥ 0.20 μg/l) [31]. Shoaibi and colleagues evaluated women and men diagnosed with AMI using standard biomarker criteria and found no variation in the assay sensitivity and specificity or troponin level by sex [32]; as well as gender, assay performance and sensitivity influenced outcome [33]. Newer “high sensitivity” clinical and preclinical cTnI and cTnT assays that do not demonstrate loss of specificity could be helpful in closing the gender bias. These new high-sensitivity assays will be expected to demonstrate robust precision and sensitivity, e.g., a highly reproducible CV < 10% at the 99th percentile concentration of the reference population, that can also be reproduced in routine practice in the clinical lab. Another important change with high-sensitivity assays will be the reporting units going to nanograms/liter instead of nanograms/milliliter (as currently reported); this is due to the at least threefold increase in sensitivity that is gained with high-sensitivity troponin assays over existing FDA-approved assays. Thus far in Europe, most high-sensitivity assays have been establishing sex-specific reference range criteria.

Overall, these data suggest that at-risk women may be missed when using male sex-specific thresholds and that, as a result, those women who meet standard MI troponin measurement criteria have suffered a greater degree of myocardial damage [33]. Indeed, a recent study demonstrated that standard troponin criteria failed to detect one out of five acute MIs occurring in women, which was, not surprisingly, associated with an elevated death rate [34], while another study demonstrated varying 99th percentiles for hs-cTnI related to lack of a uniform protocol for healthy reference population selection [35].

### High-sensitivity C-reactive protein

C-reactive protein (CRP) [10, 36, 37] is a protein synthesized in the liver in response to inflammation. Measurement of high-sensitivity CRP (hsCRP) in serum/plasma via appropriate assay formats can be used as a CVD risk marker in both men and women [38]. The concentration of hsCRP in plasma is on average higher in women (by potentially up to 60%) compared to men with and without CVD risk factors [37], a difference that is demonstrable by adolescence, whereby girls exhibit measurable CRP elevations compared to non-significant change in boys [39, 40], suggesting hormonal modulation.

While there are hundreds of papers discussing the use of hsCRP in diagnosis/prognosis of CVD and related disorders, a selection of recent presentative papers is highlighted here focusing on use in women and men. In the setting of ACS, CRP is a relatively better prognostic predictor for women compared to men [41], yet CRP is not currently used in the acute setting for decision-making for MI, not even as part of a multimarker panel. Although CRP levels were found to be lower in women than in men with HF in a prior report by Meyer et al., the women included in their study had a lesser number of comorbidities compared to the men, which may have been reflected in the lower CRP levels observed [42].

A significant relationship between hsCRP and body mass index (BMI) has been described in healthy women (*P* = 0.002), while no significant correlation was found in healthy men (*P* = 0.09) [36]. To understand this, Cartier et al. compared women and men with similar visceral and subcutaneous adiposity, respectively. They found that subcutaneous and not visceral adiposity explained the higher CRP levels in women [42]. Accordingly, given the known sex difference of higher subcutaneous adipose tissue in women compared to men [37] and the link between subcutaneous adipose tissue and CRP levels, it is not surprising to find significant sex differences in hsCRP levels despite matching for age and BMI [37]. Additionally, the finding of higher CRP in diabetic women, in particular with no known CVD, may further support its use in the “at-risk” female population [43]. This finding is further supported by a 2016 study by Garcia et al. which concluded that statistically significantly higher hsCRP levels were observed in women vs. men with metabolic syndrome risk factors, suggesting that hsCRP can be used to help stratify CVD risk even before type 2 diabetes develops [44].

Overall, these data suggest that greater use of CRP determination in the setting of ACS risk could lead to better stratification in women, as also evidenced in prior work [41].

### Brain natriuretic peptide

B-type natriuretic peptide (BNP) and atrial natriuretic peptide (ANP) are cardiac hormones secreted by the myocardium in response to excessive stretching of cardiomyocytes and are involved in hemodynamic regulation. BNP is a biologically active 32-amino acid polypeptide which functions to decrease systemic vascular resistance and increase natriuresis in times of excessive cardiac stretch. BNP is the active cleavage product of proBNP (108 aa), which is in turn produced by cleavage of the preprohormone preproBNP (134 aa precursor). BNP has been used as a biomarker for cardiac dysfunction and HF for years [45]. NT-proBNP is the remaining 76-amino acid cleavage product of proBNP and has also been used as a marker of HF even though it is not the physiologically active hormone. The half-lives of the natriuretic peptides are normally in the following order ANP < BNP < NT-proBNP, which is why BNP and NT-proBNP are measured in HF due to their longer half-lives. Additionally, owing to half-life and cleavage differences, the reference intervals for BNP and NT-proBNP are naturally quite different regardless of sex. Studies in healthy and diseased subjects have shown that BNP levels are significantly higher in women compared to men; however, cut points for this sex-based difference have not been conclusively established [24, 46–48], and it has also been observed that BNP concentration is correlated to age [49]. When comparing two commercial assays, plasma BNP was found to be 32% higher in women than in men by Shionogi and Co. assay and 80% higher by Biosite assay [48]. In one study, higher BNP levels in both women and men were associated with left ventricular systolic dysfunction; however, BNP was associated with left ventricular end-diastolic diameter/body surface area and atrial fibrillation only in women [46].

Although the etiology of higher circulating BNP levels in women is unknown, sex-specific modifier genes or mechanism might explain the association with female sex and increased BNP in tissue and circulating forms, which are modulated by disease status [50, 51]. Of particular interest in the animal model that recapitulates Takotsubo (Ampulla) cardiomyopathy, a rare stress-induced cardiomyopathy occurs predominantly in postmenopausal women, where the expression of BNP gene (and presumably, the protein product) was upregulated in the myocardium [52, 53]. Redfield et al. have described higher BNP levels in women on hormone replacement therapy compared to women not on therapy, suggesting that BNP production may be sensitive to estrogen regulation [48]. One randomized, controlled trial reported no sex differences in BNP levels; however, researchers used specific enrollment criteria that matched women and men to HF severity, resulting in similar BNP levels [42]. Similar to CRP, BNP is a relatively better prognostic predictor in the setting of ACS for women compared to men [41], yet BNP is not typically used clinically unless HF is suspected. However, continuing with the theme of sex as it relates to the use of BNP in HF (i.e., acute decompensated HF in this case), a 2016 study by Nakada et al. out of Japan observed that while there were no significant sex-specific differences in median plasma levels, high BNP vs. low BNP was correlated to a worse prognosis in men, but not women, highlighting that sex-specific differences in natriuretic peptides as they relate to HF are still not fully understood [54].

Normally, women have higher BNP levels; however, BNP levels rise to a lesser degree in women than in men with HF [55]. Therefore, the interpretation of BNP levels should include association with other biomarkers. For future personalized diagnosis and prognosis in ACS and HF patients, a temporal multimarker approach could be employed to monitor the functions of different biological pathways over time. For example, markers for status of cardiac damage (hs-cTnI), markers for pathophysiological status (CRP and other cytokines), and markers for cardiac dysfunction (BNP) can be monitored over time as a multi-marker panel [56–58].

On a more recent note, while both BNP and NT-proBNP have historically been used to assess congestive HF, with greater use of BNP overall, this may change with the introduction of sacubitril/valsartan which is a combination neprilysin inhibitor (i.e., sacubitril) and angiotensin receptor blocker (ARB) (i.e., valsartan). Neprilysin is also known as a neutral endopeptidase (NEP) or membrane metallo-endopeptidase (MME) that cleaves various peptides including natriuretic peptides. Use of this neprilysin inhibitor/ARB combo showed dramatic improvement in patient outcomes with systolic HF compared to angiotensin-converting enzyme inhibitor (ACE-inhibitor) enalapril alone in the PARADIGM-HF study [59]. Sacubitril/valsartan prevents degradation of BNP, and thus, its half-life and circulating concentration dramatically increases in patients on this drug. Because the half-life of BNP is extended, measurement of BNP in the lab in order to prognosticate these patients is questionable at best as the concentration does not decrease as expected. However, NT-proBNP appears to be unaffected by neprilysin inhibition, and therefore, early studies are favoring its use over BNP in the growing population of patients that will likely be moved to sacubitril/valsartan. That said, sex-specific differences in NT-proBNP in patients on sacubitril/valsartan warrant further study to potentially hone appropriate dosing and use of this drug.

### Additional biomarkers

There was insufficient literature for review regarding sex differences in the remainder of biomarkers listed in Table 2. While there are many knowledge gaps, Meyer et al. evaluated sex differences in a series of cardiovascular biomarkers in a cohort of women and men with HF (Table 3). This work demonstrated significant sex differences in the majority of studies [42] specifically, 6/8 inflammatory, 3/4 remodeling, and 1/1 atherosclerosis biomarkers were higher in men compared to women, while cardiomyocyte stretch was not significantly different. Combined with the prior literature, these data are consistent with the lower incidence of coronary heart disease and higher incidence of HFpEF observed in women compared to men. Notably, the angiogenesis biomarker, vascular endothelial growth factor (VEGF), was the only biomarker that was elevated in women, which is consistent with a prior angiogenesis clinical trial that was positive only in women, and supportive of the emerging concept that female progenitor cells may act as superior regenerative therapeutics compared to progenitor cells from males [60-62]. Recent studies have found that plasma levels of the neurotensin precursor hormone proneurotensin predict the development of cardiovascular disease and are significantly higher in women compared to men [63–65].

**Table 3:** Sex-specific biomarker levels in heart failure (reprinted with permission) [42]K

	Total cohort (*n* = 567)	Male (*n* = 351)	Female (*n* = 216)	*P* value
Inflammation				
C-reactive protein, μg/mL	11.4 (4.8–33.0)	13.0 (5.5–33.0)	9.0 (4.2–28.9)	0.018
PTX 3, ng/mL	3.7 (2.5–5.6)	3.9 (2.7–5.8)	3.3 (2.2–5.0)	0.002
GDF-15, ng/mL	2.8 (1.9–4.3)	3.1 (2.2–4.7)	2.4 (1.7–3.8)	0.000
Osteopontin, ng/mL	159.2 (109.0–223.1)	165.7 (111.4–232.8)	147.2 (100.9–209.3)	0.083
RAGE, ng/mL	2.9 (1.9–4.6)	3.0 (1.9–4.7)	2.7 (1.9–4.2)	0.165
Interleukin 6, ng/mL	12.0 (6.8–24.3)	13.1 (7.9–28.4)	10.9 (5.9–18.4)	< 0.001
TNF-α, pg/mL	45.8 (4.7–121.3)	47.3 (4.7–146.4)	43.7 (4.8–85.0)	0.230
TNF-αR1a, ng/mL	3.1 (2.2–4.6)	3.1 (2.2–4.7)	2.9 (2.2–4.4)	0.500
Oxidative stress				
MPO, ng/mL	20.1 (15.6–28.1)	20.4 (15.7–28.4)	19.1 (15.3–26.5)	0.115
Remodeling				
Syndecan-1, ng/mL	20.8 (15.4–28.5)	20.8 (15.4–28.5)	17.7 (12.2–26.1)	0.004
Periostin, ng/mL	4.7 (3.4–6.6)	5.0 (3.5–6.6)	4.4 (3.1–6.3)	0.023
Galectin 3, ng/mL	25.6 (21.1–32.1)	26.2 (21.5–32.5)	24.9 (20.2–31.2)	0.057
TGF-β, ng/mL	51 (35–75)	48 (34–72)	53 (36–82)	0.043
Cardiomyocyte stretch				
NTpro-BNP, pg/mL	2532 (1309–5721)	2677 (1407–6340)	2344 (1197–5047)	0.978
ST-2, ng/mL	2.5 (1.4–5.4)	2.6 (1.5–5.4)	2.2 (1.2–5.5)	0.069
Angiogenesis				
VEGF, ng/mL	63.0 (31.4–143.8)	58.7 (27.3–118.0)	73.1 (36.8–189.4)	0.003
Angiogenin, μg/mL	5.1 (3.6–7.5)	5.0 (3.6–7.4)	5.3 (3.5–8.0)	0.465
Arteriosclerosis				
ESAM, ng/mL	53.0 (44.5–64.3)	54.1 (45.5–65.1)	51.3 (43.0–62.1)	0.038
Renal function				
eGFR, mL/min/1.73m^2^	53.9 ± 20.2	55.8 ± 19.9	50.9 ± 20.2	0.006
Cystatin C, μg/mL	11.1 (7.6–16.2)	11.1 (7.7–16.9)	11.1 (7.6–15.7)	0.774
NGAL, ng/mL	84.6 (60.4–123.3)	85.8 (61.3–135.9)	83.8 (58.8–116.1)	0.127
Anemia				
Hb, g/dL	13.1 ± 2.0	13.4 ± 2.1	12.6 ± 1.8	< 0.001
EPOa, IU/L	9.6 (5.2–16.0)	9.7 (5.1–16.5)	9.5 (5.2–15.0)	0.569

*PTX3* pentraxin 3, *GDF-15* growth differentiation factor 15, *RAGE* receptor for advanced glycation end products, *TNF-α* tumor necrosis factor alpha, *TNF-αR1a* tumor necrosis factor alpha receptor 1a, *MPO* myeloperoxidase, *TGF-β* transforming growth factor-beta, *NTpro-BNP* N-terminal pro-brain natriuretic peptide, *ST-2* suppression of tumorigenicity 2, *VEGF* vascular endothelial growth factor, *EPOa* erythropoietin alpha, *ESAM* endothelial cell-selective adhesion molecule, *NGAL* neutrophil gelatinase-associated lipocalin

## Conclusions

Use of one or more cardiac biomarkers can help physicians identify IHD and/or HF and initiate appropriate therapy for both women and men. While the focus of cardiac biomarker studies in previous decades primarily focused on men, the number of women with IHD and HF has increased, and women overall now account for the majority of cardiovascular disease-related deaths [6]. Recent work suggests that there may be a diagnosis gap, whereby more men are diagnosed and treated for cardiovascular disease compared to women, potentially contributing to higher female mortality rates [7, 34].

Our review highlights the well-described sex differences in multiple IHD and HF biomarkers clinically used on a daily basis to diagnose and treat women and men [13, 22–24, 29–33, 42, 46, 47, 53]. This includes the gold-standard troponins (cTnI and cTnT) and its lesser used and useful alternative, CK-MB, which exhibit 99th percentile thresholds that are significantly lower in women than men, yet male standards are still in widespread use; this disparity is a primary contributing factor in failure to accurately and adequately diagnose ischemia and myocardial infarction in women. Additional biomarkers, hsCRP and BNP, are more often elevated and prognostically more useful in women compared to men, yet they are less often clinically employed for risk stratification.

Mounting evidence indicates that the failure to use sex-specific biomarker strategies may contribute to the CVD diagnosis mortality gap, whereby fewer women are being diagnosed but more are dying from CVD. Accurate diagnosis, appropriate management of risk, and ongoing monitoring are key in the prevention and treatment of CVD [66]. Indeed, diagnostic uncertainty portends therapeutic uncertainty and missed treatment opportunities.

The described sex differences in CVD biomarkers reviewed herein suggest that the diagnosis and management of CVD may be optimally personalized for women and men by the use of sex-specific biomarker thresholds in clinical laboratory reporting. This should assist physician recognition of “female-pattern” CVD and reduce the number of false negatives in at-risk women. Appropriate recognition of CVD based on well-defined biomarker sex thresholds will allow for tailored and timely treatment, which will naturally lead to improved outcomes for both sexes. It is time to acknowledge that CVD presents differently not only symptomatically, but also biochemically, in women and men, and future research should focus on identifying these differences and developing sex-specific diagnostic and prognostic guidelines where appropriate.

## Abbreviations

ACS: Acute coronary syndrome
AHA/ACC: American Heart Association/American College of Cardiology
AMI: Acute myocardial infarction
ANP: Atrial natriuretic peptide
ARB: Angiotensin receptor blocker
BMI: Body mass index
BNP: B-type natriuretic peptide
CK: Creatine kinase
CKB: Creatine kinase brain
CKM: Creatine kinase muscle
CRP: C-reactive protein
cTn: Cardiac troponins
cTnI: Cardiac troponin I
cTnT: Cardiac troponin T
CVD: Cardiovascular disease
HFpEF: Heart failure (HF) with preserved ejection fraction
hsCRP: High-sensitivity C-reactive protein
IFCC: International Federation of Clinical Chemistry
IHD: Ischemic heart disease
MI: Myocardial infarction
MME: Membrane metallo-endopeptidase
NACB: National Academy of Clinical Biochemistry
NEP: Neutral endopeptidase
NIH: National Institutes of Health
NSTEMI: Non-ST segment MI
NT-proBNP: N-terminal pro b-type natriuretic peptide
VEGF: Vascular endothelial growth factor
